# Ubiquitin-specific peptidase 25 exacerbated osteoarthritis progression through facilitating TXNIP ubiquitination and NLRP3 inflammasome activation

**DOI:** 10.1186/s13018-023-04083-y

**Published:** 2023-10-09

**Authors:** Jie Sui, Fei Dai, Jiusheng Shi, Changcheng Zhou

**Affiliations:** Department of Orthopedics, 904 Hospital of PLA Joint Logistic Support Force, 55 Heping North Road, Changzhou, 213003 Jiangsu Province China

**Keywords:** Osteoarthritis, USP25, TXNIP, Ubiquitination, NLRP3 inflammasome, ROS

## Abstract

Several members of the ubiquitin-specific proteases (USPs) family have been revealed to regulate the progression of osteoarthritis (OA). The current study aimed to investigate the role and the underlying mechanism of USP25 in IL-1β-induced chondrocytes and OA rat model. It was discovered that IL-1β stimulation upregulated USP25, increased ROS level, and suppressed cell viability in rat chondrocytes. Besides, USP25 knockdown alleviated IL-1β-induced injury by decreasing ROS level, attenuating pyroptosis, and downregulating the expression of IL-18, NLRP3, GSDMD-N, active caspase-1, MMP-3, and MMP-13. Furthermore, we discovered that USP25 affected the IL-1β-induced injury in chondrocytes in a ROS-dependent manner. Moreover, USP25 was revealed to interact with TXNIP, and USP25 knockdown increased the ubiquitination of TXNIP. The pro-OA effect of USP25 abundance could be overturned by TXNIP suppression in IL-1β-induced chondrocytes. Finally, in vivo experiment results showed that USP25 inhibition alleviated cartilage destruction in OA rats. In conclusion, we demonstrated that USP25 stimulated the overproduction of ROS to activate the NLRP3 inflammasome via regulating TXNIP, resulting in increased pyroptosis and inflammation in OA.

## Introduction

Osteoarthritis (OA), a common degenerative joint disease [1], is characterized by synovial inflammation, cartilage and ligaments degeneration, osteophytes formation, and subchondral bone impairment [2]. Several risk factors, including aging, obesity, abnormal metabolism, joint injury, and heredity, have been reported to contribute to OA initiation and progression [3], and the current therapies for OA mainly focus on alleviating inflammatory pain and knee stiffness but are ineffective in OA prevention or treatment [4]. Therefore, it is still urgent to explore potential therapeutic approaches for OA.

Ubiquitin-specific peptidase 25 (USP25) is a deubiquitinating enzyme belonging to the USPs family [5]. Multiple USP family members have been reported to mediate OA progression. For example, USP3 was deficient in OA and USP3 abundance suppressed IL-1β-induced chondrocyte apoptosis by targeting TRAF6 [6]. USP49 facilitated the deubiquitination of Axin and suppressed IL-1β-stimulated apoptosis of chondrocytes by inhibition of Wnt/β-catenin signaling [7]. USP13 upregulated PTEN expression and restrained oxidative stress, apoptosis, and inflammation in OA via AKT signaling [8]. Abundant USP7 accelerated the progression of H_2_O_2_-treated chondrocytes injury through NOX4/NLRP3 pathway [9]. USP25 has been proved to play either pro-inflammatory or anti-inflammation role in different conditions [5, 10, 11]. Noticeably, USP25 upregulation was reported to boost NLRP3 inflammasome-mediated pyroptosis of acinar cells in acute pancreatitis [12]. Therefore, we speculated that USP25 may mediate inflammation in OA, probably through its interaction with the NLRP3 inflammasome.

NLRP3 inflammasome is a multi-protein complex and consists of NLRP3, ASC, and pro-caspase-1 [13]. Aberrant activity of NLRP3 inflammasome has been reported to be associated with the pathogenesis of various diseases, such as gout, diabetes, and Alzheimer’s disease [14]. Recently, the activation and participation of NLRP3 inflammasome in OA progression have been increasingly reported. For example, Chen et al. uncovered that the activation of NLRP3 inflammasome could be enhanced by inhibiting Nrf2/HO-1 in OA [15]. Zu et al. reported that Icariin alleviated OA development through the suppression of NLRP3/caspase-1 signaling [16]. Zhang et al. proved that NLRP3 inflammasome was activated in OA and inhibition of NLRP3 inflammasome by CY-09 repressed OA development [17]. In addition, NLRP3 inflammasome was activated by thioredoxin-interacting protein (TXNIP) in many cases [18–20]. Nevertheless, it is unclear whether USP25 regulates OA progression via TXNIP/NLRP3 pathway.

This investigation aims to elucidate the effect of USP25 on the progression of OA and the molecular mechanism involved. It was found that USP25 exacerbated OA progression through the TXNIP/NLRP3 pathway, indicating USP25 as a potent therapeutic biomarker in OA treatment.

## Materials and methods

### Isolation and culture of rat chondrocytes

Rat chondrocytes were collected following previous steps [21]. Isolated chondrocytes were cultured in DMEM/F12 containing 10% fetal bovine serum (FBS, Gibco), 100 U/ml penicillin and streptomycin (Sigma-Aldrich) at 37 ℃ with 5% CO_2_. For IL-1β induction, chondrocytes were subjected to IL-1β (10 ng/ml, R&D System) for 24 h.

### Cell transfection

USP25 overexpression plasmid (pcDNA3.1/USP25) was constructed by inserting USP25 cDNA fragment into pcDNA3.1 vector and empty vector was used as control. USP25 shRNA (shUSP25) and TXNIP shRNA (shTXNIP) and their control (shNC) were synthesized by Shanghai Genepharma. The overexpression plasmids and shRNAs were transfected into rat chondrocytes with Lipofectamine 3000 (Invitrogen).

### ELISA assay

The concentration of IL-18, MMP-3, and MMP-13 in the supernatants of chondrocytes was detected using ELISA Kit (R&D System) as per the supplier’s instructions.

### RT-qPCR

RNA was extracted with TRIzol Reagent (Invitrogen). RevertAid First Strand cDNA Synthesis kit was used for cDNA synthesis. Quantification of mRNA levels was conducted using SYBR Green polymerase chain reaction (PCR) Mix (Thermo Fisher) on ABI Prism 7300 SDS System (Applied Biosystem). GAPDH was used as internal control. The primer sequences used were USP25-forward, 5′-CCCTACCATCACAGTCCTTACC-3′; USP25-reverse, 5′-CTGGAGGTATCCGAGACTGAGT-3′; TXNIP-forward, 5′-CAGCAGTGCAAACAGACTTCGG-3′; TXNIP-reverse, 5′-CAGCAGTGCAAACAGACTTCGG-3′; GAPDH-forward, 5′-GTCTCCTCTGACTTCAACAGCG-3′; and GAPDH-reverse, 5′-ACCACCCTGTTGCTGTAGCCAA-3′.

### Western blot

Western blot was performed following steps previously described [22]. Briefly, proteins were isolated from cartilage tissues of experimental rats or chondrocytes with RIPA lysis buffer and quantified using BCA protein kit (Both from Beyotime). 10 µg protein/lane was separated by 10% SDS-PAGE and transferred onto PVDF membranes. Subsequently, the membranes were incubated with primary antibodies against USP25, NLRP3, GSDMD-N, active caspase-1, TXNIP, and GAPDH at 4 °C overnight, followed by an incubation with horseradish peroxidase-conjugated secondary antibody for 1 h. Membranes were visualized with chemiluminescence reagent (Bio-Rad) with a ChemiDoc imaging machine (Bio-Rad).

### Cycloheximide-chase assay

IL-1β-challenged chondrocytes were transfected with shNC or shUSP25. 48 h after transfection, the original medium was replaced with the fresh medium containing Cycloheximide (10 μg/ml). Then, the expression of TXNIP was determined using the western bolt at 0, 3, 6, 9 h after Cycloheximide treatment.

### CCK-8 assay

Cell Counting Kit 8 (CCK-8, Dojindo) was used to assess the proliferation of chondrocytes. Cells were seeded into 96-well plates and CCK-8 reagent (10 μL) was added at indicated time points (0, 24, 48, 72 h). Cell viability was determined by measuring the optical density at 450 nm (OD 450 nm) with a microplate reader (Bio-Rad).

### Detection of pyroptosis

The pyroptosis of chondrocytes was assessed using caspase-1 Detection Kit (ImmunoChemistry) following manufacturer’s instructions. Briefly, cells were incubated with FAM-YVAD-FMK in the dark and then resuspended in PI staining buffer. Pyroptosis was defined as double positive with FAM-YVAD-FMK and PI staining and determined by flow cytometer (BD Biosciences).

### Detection of ROS levels

ROS production was detected using DCFH-DA provided in the reactive oxygen species assay kit (Beyotime Biotechnology) as per supper’s manual. Briefly, rat chondrocytes were incubated with DCFH-DA for 20 min at 37 °C in the dark. Relative fluorescent intensity was analyzed using flow cytometry with excitation wavelength at 488 nm and emission wavelength at 525 nm.

### Co-immunoprecipitation (Co-IP) and ubiquitination assay

Co-IP and ubiquitination assay was performed as previously described [23]. Cell lysates were extracted with RIPA buffer and incubated with specific antibodies at 4 °C overnight. Subsequently, the mixture of protein and antibodies was incubated with protein A/G beads (Santa Cruz) at 4 °C for 4 h. Bound protein was detected by western blot. For ubiquitination assay, the cells were treated with Mg132 and then lysed by SDS-free RIPA buffer and immunoprecipitated with indicated antibodies followed by A/G plus agarose. The supernatant was analyzed by western blot using ubiquitin antibody.

### Establishment of OA model in rats

Male Sprague–Dawley rats (200 ± 20 g) were obtained from the Animal Center of Chinese Academy of Sciences (Shanghai, China). The OA model was established by injection of monosodium iodoacetate (MIA) into the knee joint cavities of experimental rats as previously described [24]. The rats were randomly divided into three groups: Control, MIA, and MIA + AZ1. For AZ1 treatment, AZ1 was dissolved in DMSO (100 mM, 42.2 mg ml^−1^) to make a stock. AZ1 was diluted in 100–200 μl of PBS and administered to the rats through intragastric gavage (20 mg/kg). After 6 weeks of indicated treatments, the knee joint tissues were collected for further experiments.

### Histological analysis

To analyze the histological changes, knee joint tissues were embedded in paraffin and then cut into Sections (5 μm) using a rotary microtome. Then, the sections were stained with hematoxylin–eosin (H&E) and Safranin O to be observed under a light microscope. Cartilage destruction was examined by Safranin O staining and the score was assessed using the Osteoarthritis Research Society International (OARSI) grading system.

## Results

### IL-1β stimulation upregulated USP25, increased ROS level and hindered the viability of chondrocytes

**Fig. 1:**
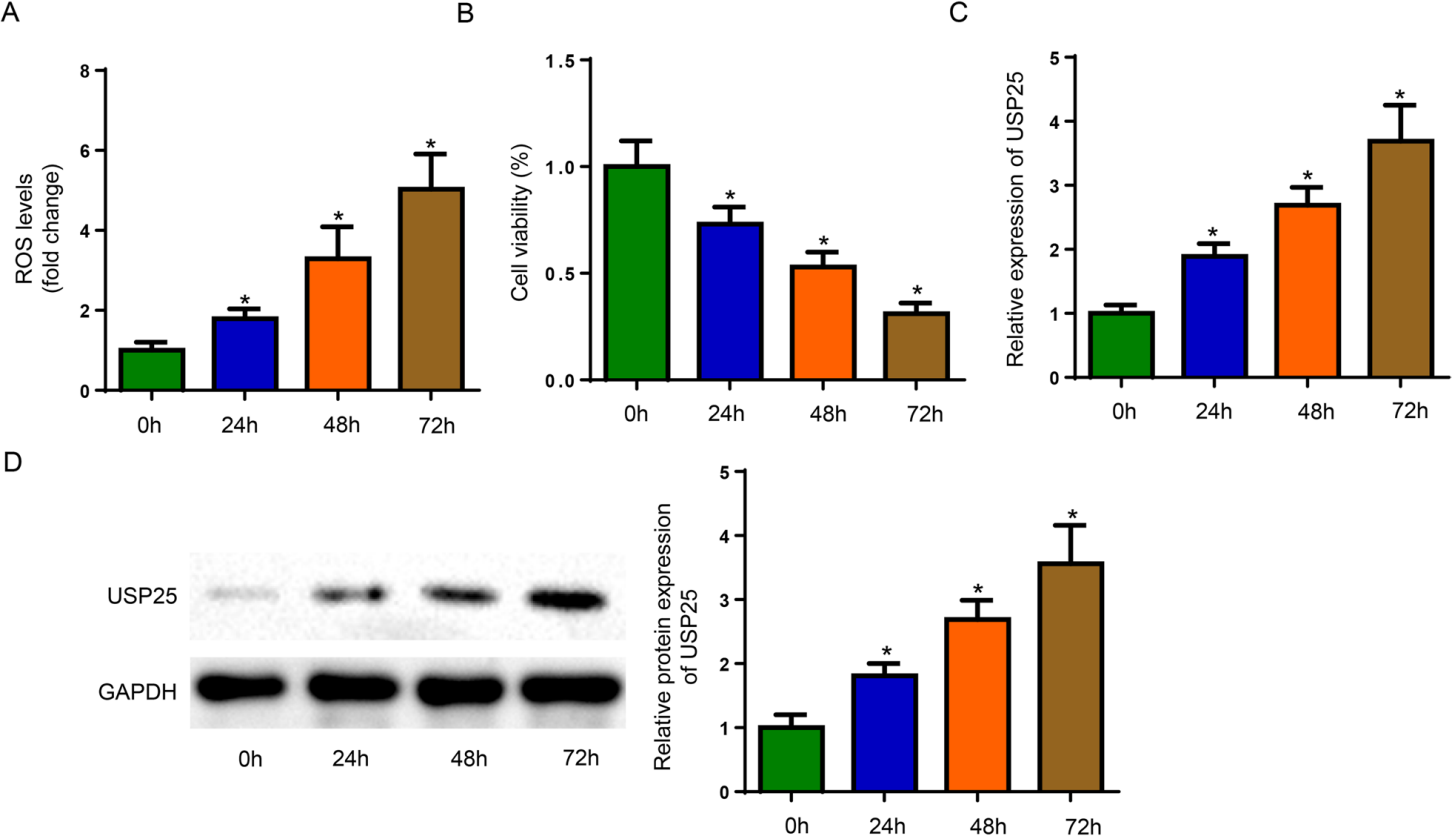
IL-1β-treated rat chondrocytes exhibited higher USP25 expression, elevated ROS level, and impaired cell viability. The isolated chondrocytes were treated with IL-1β for 0, 24, 48, 72 h. **A** The ROS production was evaluated by flow cytometry. **B** Cell proliferation was determined using CCK-8 assay. **C** and **D** The protein and mRNA expression of USP25 was detected by western blot and RT-qPCR, respectively. *P < 0.05

Firstly, the effect of IL-1β on the injuries of isolated rat chondrocytes was investigated. The chondrocytes were treated with IL-1β for different time points (0, 24, 48, 72 h), and the ROS production, cell proliferation, and USP25 expression were detected. As shown in Fig. 1A–D, the ROS level and USP25 expression were gradually increased and cell proliferation was gradually decreased in a time-dependent manner. These results suggested that USP25 might be involved in IL-1β-induced ROS production and cell viability suppression.

### USP25 knockdown attenuated IL-1β-stimulated pyroptosis, ROS level, ECM degradation, and activation of NLRP3 inflammasome

**Fig. 2:**
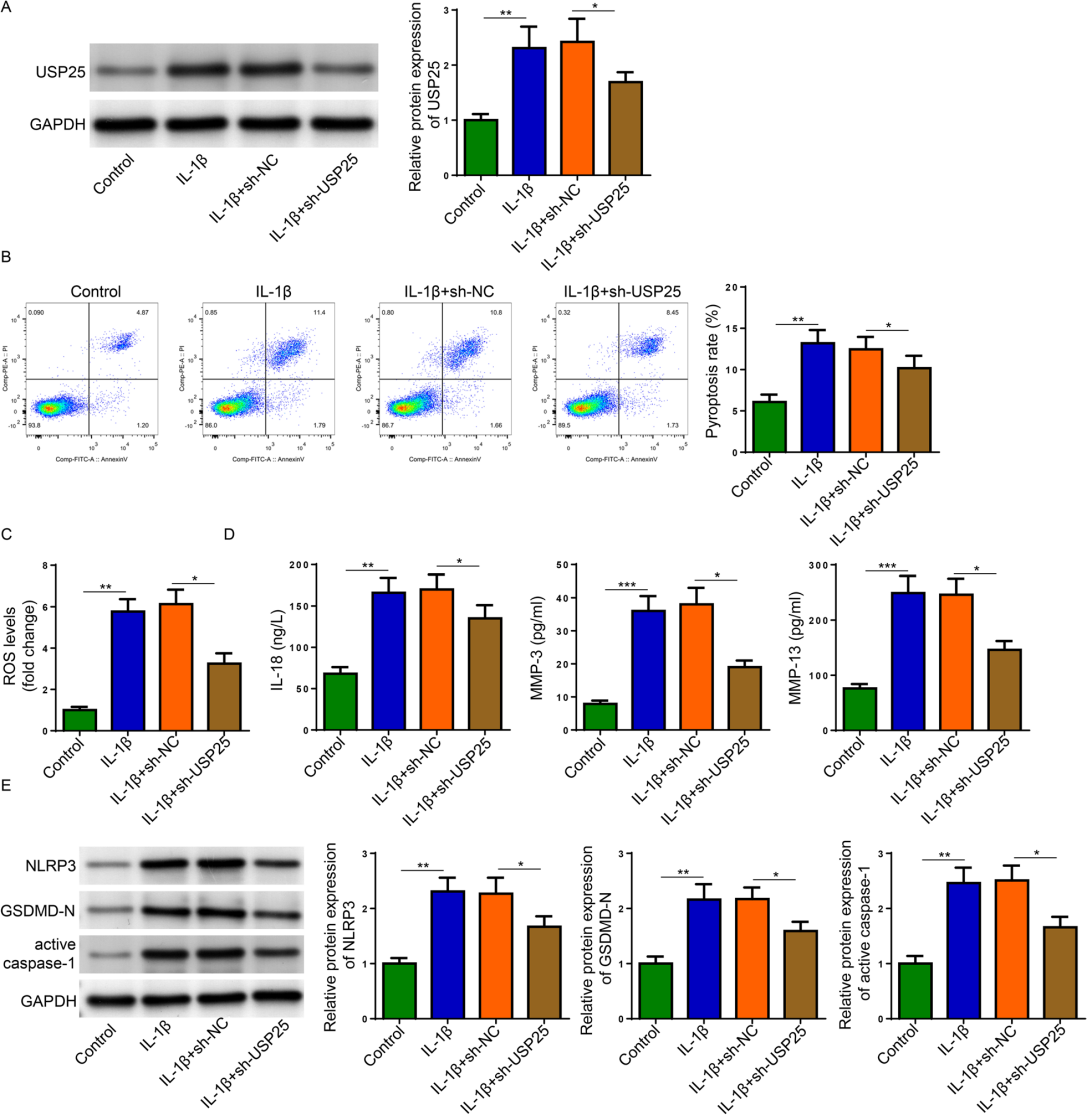
USP25 ameliorated IL-1β-induced injury in rat chondrocytes. The chondrocytes were treated by IL-1β and then transfected with shUSP25. **A** The protein expression of USP25 was detected Western blot. **B**, **C** Pyroptosis and ROS level were detected by Flow Cytometry. **D** ELISA assay detected the expression of IL-18, MMP-3, and MMP-13 in cell supernatants. **E** Western blot detected the protein levels of NLRP3, GSDMD-N, active caspase-1. *P < 0.05; **P < 0.01; ***P < 0.001

To evaluate the biological role of USP25, USP25 was successfully silenced in IL-1β-treated OA chondrocytes as indicated by western blot (Fig. 2A). Flow cytometry indicated that USP25 depletion reversed the increase of pyroptosis and ROS production caused by IL-1β stimulation (Fig. 2B and C). ELISA results showed that IL-1β-induced upregulation of pro-inflammatory cytokine (IL-18) and MMPs (MMP-3 and MMP-13) were suppressed by USP25 depletion (Fig. 2D). The protein levels of NLRP3, GSDMD-N, and active caspase-1 were markedly elevated in IL-1β-treated cells, which were decreased by silencing USP25 (Fig. 2E). To sum up, USP25 depletion alleviated IL-1β-induced pyroptosis, ROS production, ECM degradation, and activation of NLRP3 inflammasome.

### USP25 promoted pyroptosis and activated NLRP3 inflammasome in IL-1β-induced chondrocytes by stimulating ROS production

**Fig. 3:**
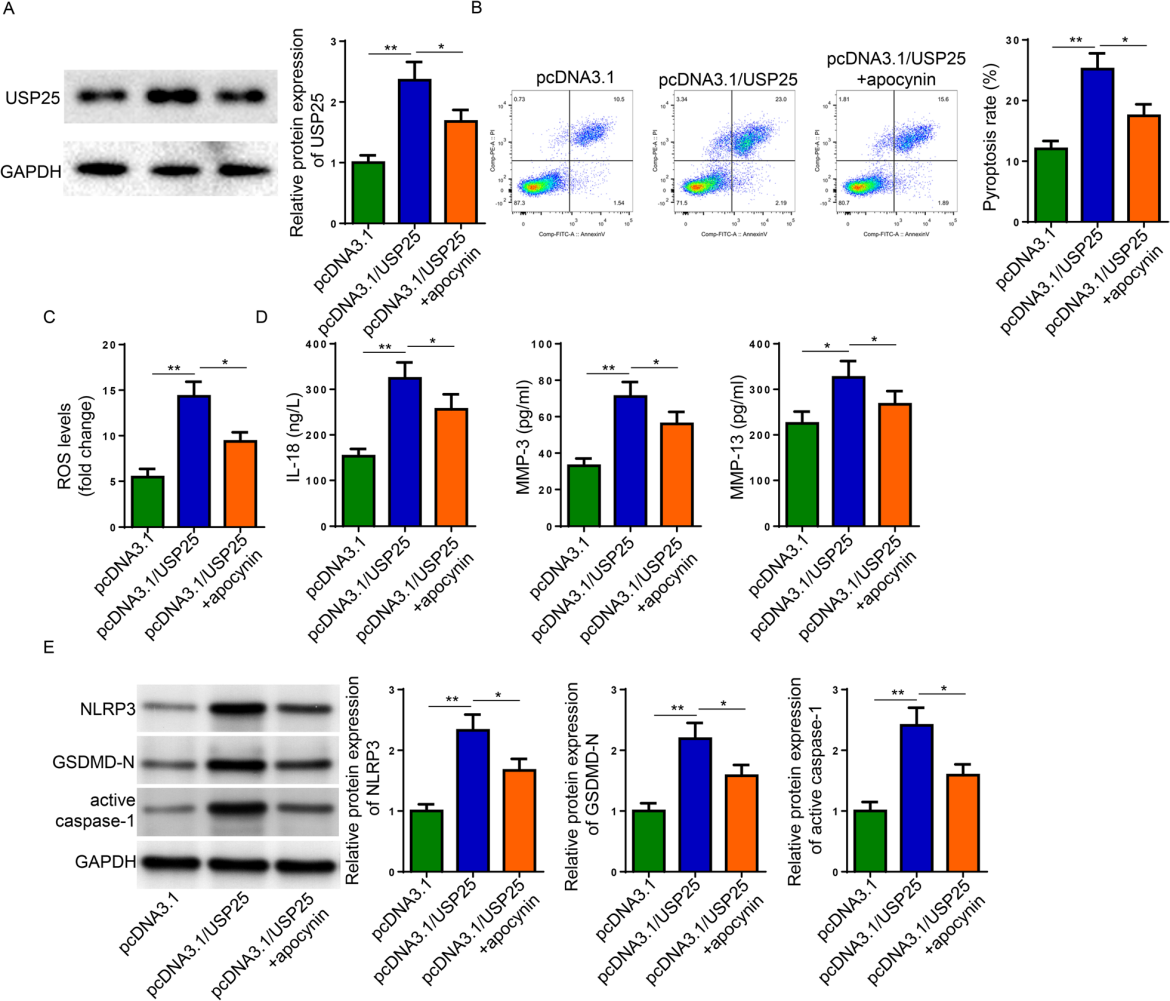
USP25 exacerbated IL-1β-induced injury in chondrocytes in ROS-dependent manner. The IL-1β-induced chondrocytes were treated with pcDNA3.1, pcDNA3.1/USP25 or pcDNA3.1/USP25 + apocynin. **A** The protein expression of USP25 was detected Western blot. **B**, **C** Pyroptosis and ROS level were detected by Flow Cytometry. **D** ELISA assay detected the expression of IL-18, MMP-3, and MMP-13 in cell supernatants. **E** Western blot detected the protein levels of NLRP3, GSDMD-N, active caspase-1. *P < 0.05; **P < 0.01

To investigate the role of ROS in USP25-mediated pyroptosis and NLRP3 inflammasome activation, apocynin (ROS inhibitor) was used in subsequent studies. Firstly, western blot confirmed the successful overexpression of USP25 and the participation of apocynin partially reversed the effect of USP25 overexpression (Fig. 3A). Flow cytometry analysis indicated that apocynin decreased the pyroptosis and ROS production of IL-1β-treated chondrocytes induced by USP25 abundance (Fig. 3B and C). Besides, the expression of IL-18, MMP-3, MMP-13, NLRP3, GSDMD-N, and active caspase-1 increased by USP25 overexpression was decreased by apocynin (Fig. 3D and E). Taken together, USP25 increased ROS production to exacerbate IL-1β-induced injuries in OA chondrocytes.

### USP25 suppressed the ubiquitination of TXNIP in IL-1β-treated chondrocytes

**Fig. 4:**
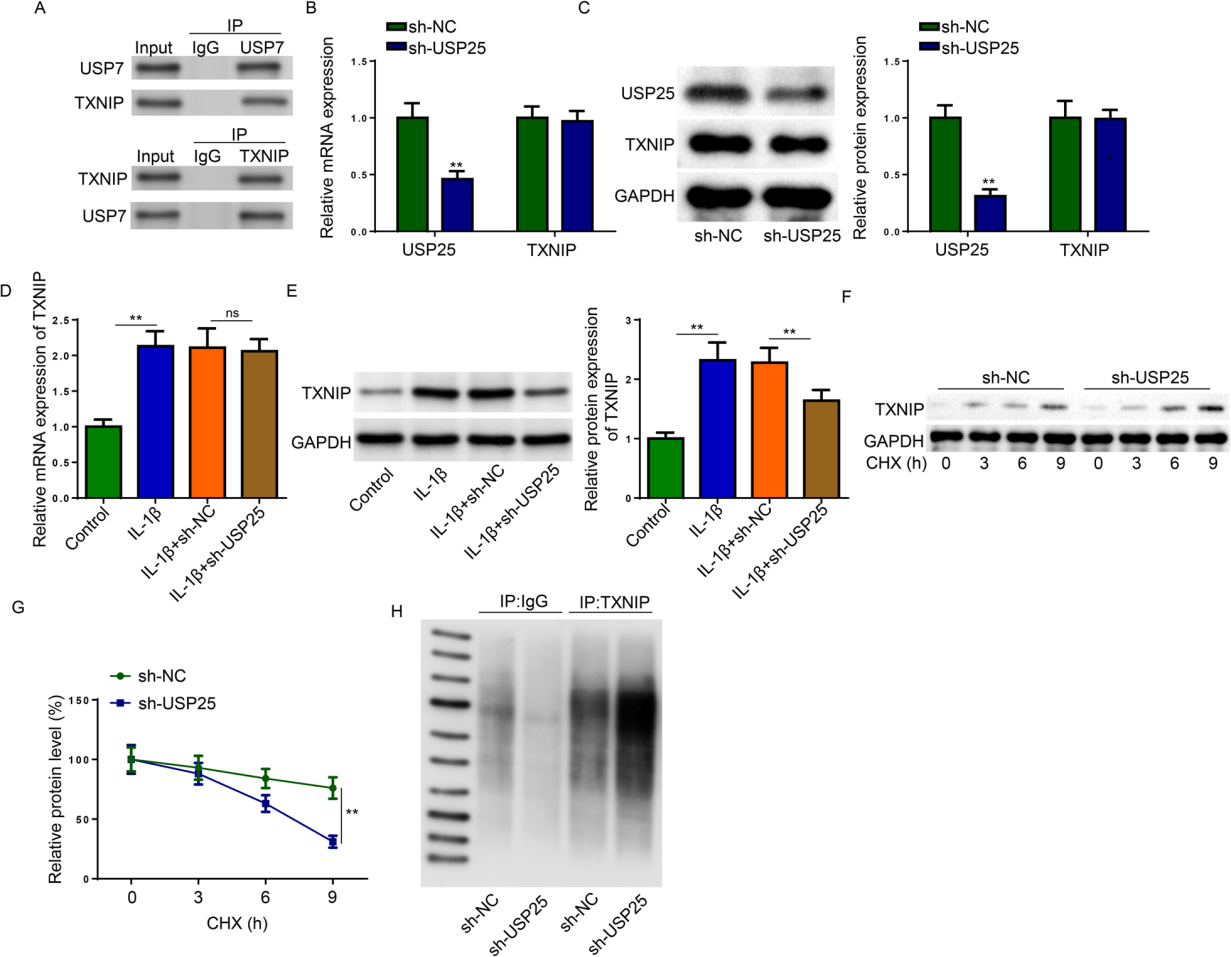
USP25 interacted with TXNIP in IL-1β-induced chondrocytes. **A** Co-immunoprecipitation was performed with IgG, and antibodies against USP25 and TXNIP. **B** The mRNA expression of USP25 and TXNIP in chondrocytes transfected with shNC or shUSP25 were detected. **C** The protein expression of USP25 and TXNIP in chondrocytes transfected with shNC or shUSP25 were detected. **D** The mRNA expression of USP25 and TXNIP in IL-1β-induced chondrocytes transfected with shNC or shUSP25 were detected. **E** The protein expression of TXNIP in IL-1β-induced chondrocytes silenced with USP25. **F** Representative western blotting for TXNIP in IL-1β-induced chondrocytes transfected with shNC or shUSP25 subjected to CHX pulse-chase assay **G** and densitometric quantification of TXNIP. **H** USP25-silenced chondrocytes were immunoprecipitated with TXNIP or IgG antibodies and ubiquitination was assessed by western blot. **P < 0.01

DUBs modulate biological activities by affecting the degradation or function of substrate proteins [25]. Previous studies showed that TXNIP was associated with ROS production and the activation of NLRP3 inflammasome in OA [26, 27]. Besides, reinforced ubiquitination has been proved to cause decreased TXNIP expression [28, 29]. Therefore, we speculated that the deubiquitinase USP25 might interact with TXNIP and promoted its expression through inhibiting ubiquitylation. A Co-IP assay first confirmed that USP25 bind with TXNIP in IL-1β-treated chondrocytes (Fig. 4A). To verify whether USP25 has a regulatory role in TXNIP, USP25 was silenced in chondrocytes, which substantially reduced the mRNA and protein expression of USP25 but had no significant effect on the level of TXNIP (Fig. 4B and C). Interestingly, the protein expression but not the mRNA expression of TXNIP was decreased by shUSP25 in IL-1β-treated chondrocytes (Fig. 4D and E), suggesting that USP25 deficiency might facilitate the intracellular degradation of TXNIP in 1β-treated chondrocytes. Expectedly, the rate of TXNIP protein degradation was significantly increased in USP25-silenced chondrocytes when cycloheximide was added to prevent new protein synthesis (Fig. 4F and G). Moreover, ubiquitination assay results showed that USP25 knockdown significantly increased the ubiquitination of TXNIP (Fig. 4H). These results suggested that USP25 binds with TXNIP in IL-1β-treated chondrocytes and the knockdown of USP25 decreased TXNIP expression by reinforcing its ubiquitination.

### USP25 deubiquitinated TXNIP to promote pyroptosis, ROS production, and NLRP3 inflammasome activation in IL-1β-induced chondrocytes

**Fig. 5:**
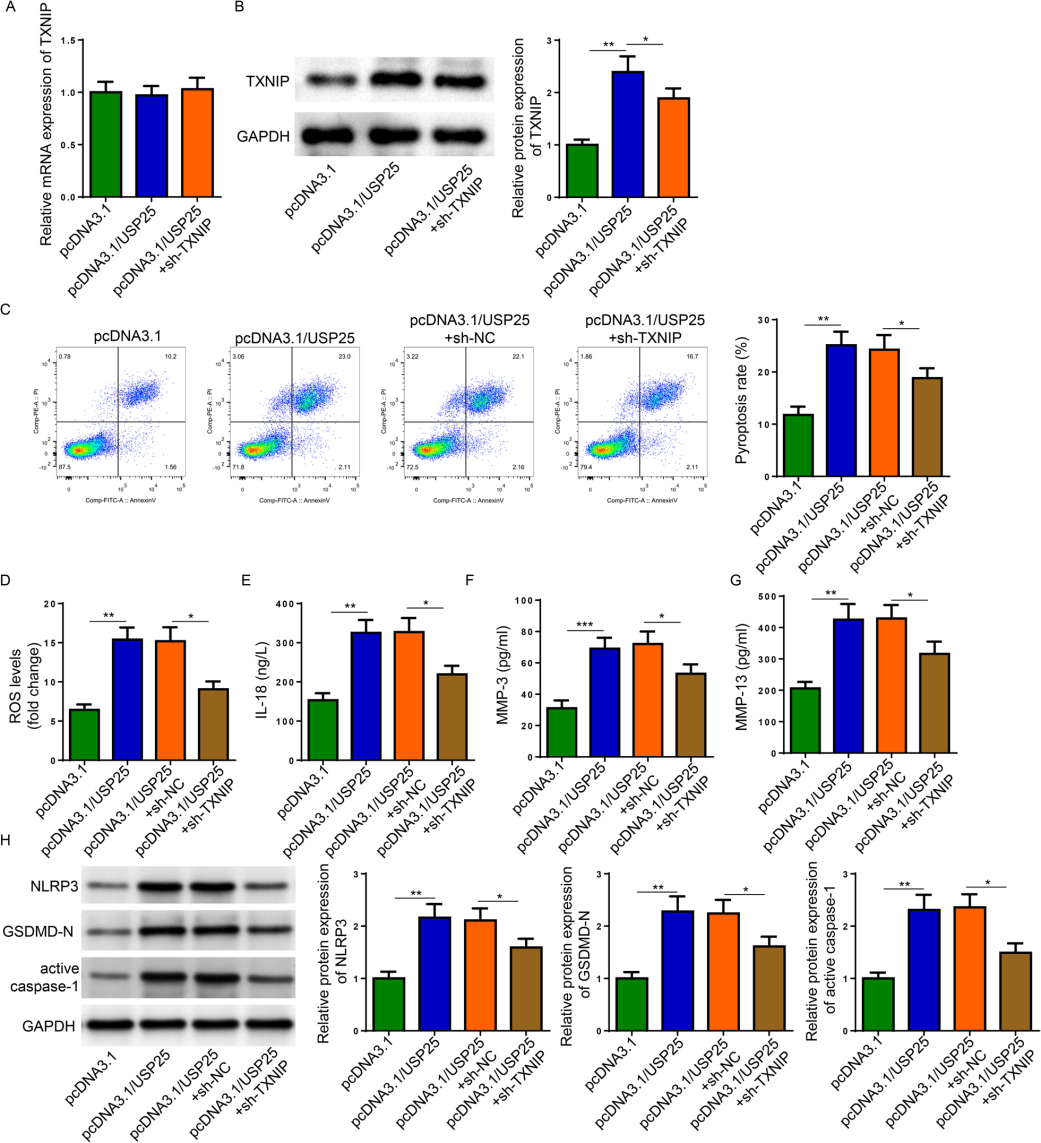
USP25 mediated the IL-1β-induced injury in chondrocytes by interacting with TXNIP. IL-1β-induced chondrocytes were transfected with pcDNA3.1, pcDNA3.1/USP25 or pcDNA3.1/USP25 + shTXNIP. **A**, **B** The mRNA and protein expression of TXNIP was detected. **C**, **D** Pyroptosis and ROS level were detected by Flow Cytometry. **E**–**G** ELISA assay detected the expression of IL-18, MMP-3, and MMP-13 in cell supernatants. (**H**) Western blot detected the protein levels of NLRP3, GSDMD-N, active caspase-1. *P < 0.05; **P < 0.01; ***P < 0.001

The biological role of the interaction between USP25 and TXNIP was further investigated by overexpressing USP25 while silencing TXNIP in IL-1β-induced chondrocytes (Fig. 5A and B). Flow cytometry results revealed that inhibition of TXNIP reversed the promotive effect of USP25 overexpression on pyroptosis and ROS production (Fig. 5C and D). In addition, TXNIP deficiency abrogated the increase in levels of IL-18, MMP-3, MMP-13, NLRP3, GSDMD-N, and active caspase-1 resulted from USP25 abundance in the synovial tissues of knee joint (Fig. 5E-H). To summarize, USP25 interacted with TXNIP to affect IL-1β-induced injuries in OA chondrocytes.

### USP25 knockdown retarded OA progression in vivo

**Fig. 6:**
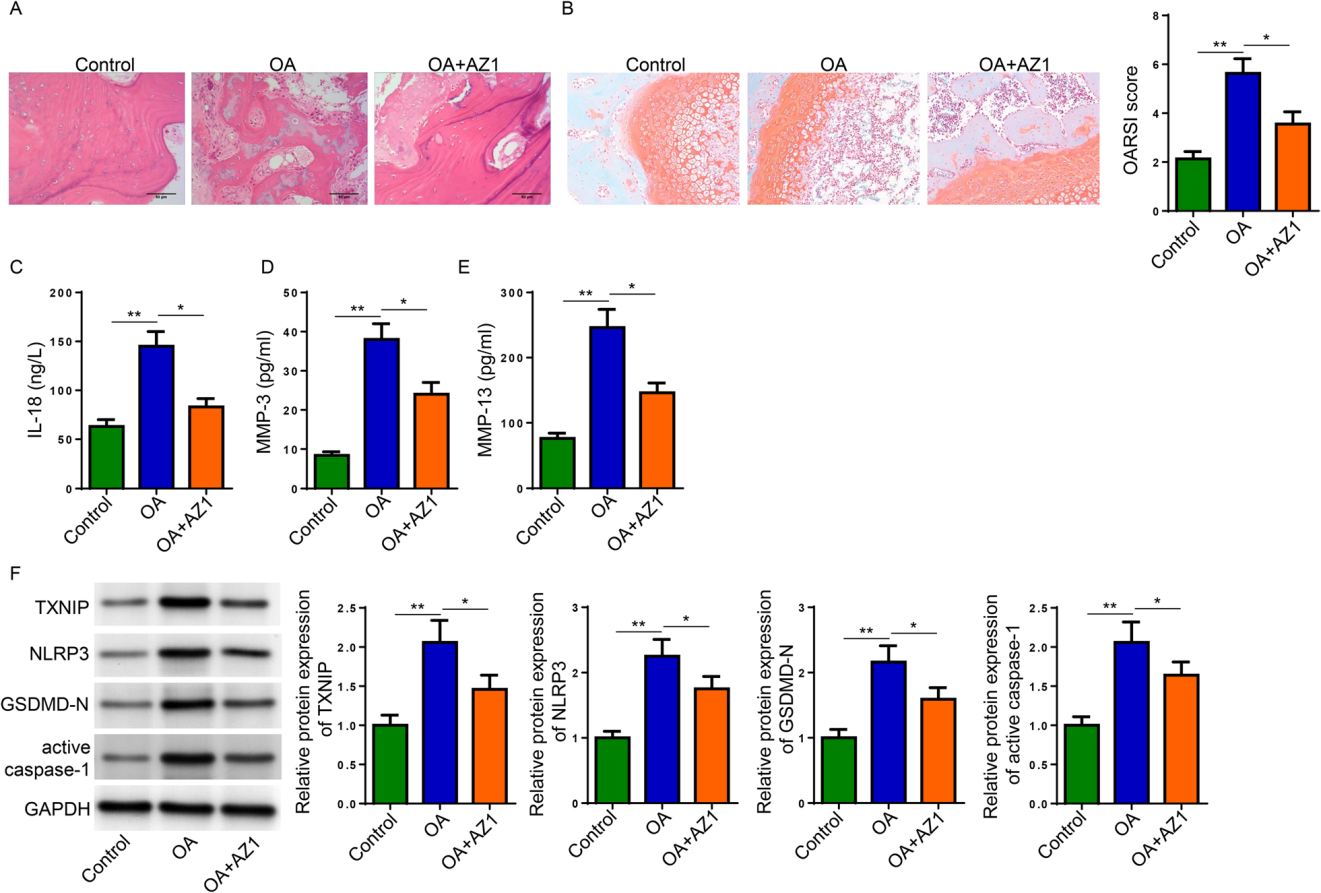
USP25 knockdown attenuated OA progression in vivo. Rats were injected with MIA to establish an OA model with or without AZ1 treatment. **A** Representative H&E images of knee joint tissues from experimental rats. **B** Safranin O staining images of mice knee joint tissues and OARSI scores of each group to assess cartilage degeneration. **C**–**E** IL-18, MMP-3, and MMP-13 levels, and **F** protein levels of TXNIP, NLRP3, GSDMD-N, active caspase-1. *P < 0.05; **P < 0.01

Afterwards, we investigated the effect of USP25 on OA rat model by using USP25 inhibitor (AZ1). H&E and Safranin O staining indicated that MIA-induced damage in articular tissues was effectively ameliorated by AZ1 administration (Fig. 6A and B). Next, IL-18, MMP-3, and MMP-13 concentration in the synovial fluid was measured by ELISA. The results showed that AZ1 administration caused a significant decrease in the upregulated concentration of IL-18, MMP-3, and MMP-13 in MIA-challenged rats (Fig. 6C-E). Additionally, western blot detected that MIA-induced increased levels of TXNIP, NLRP3, GSDMD-N, active caspase-1 were attenuated by the inhibition of USP25 with AZ1 in cartilage tissues of experimental rats (Fig. 6F). To conclude, USP25 inhibition suppressed OA progression in vivo.

## Discussion

OA, a progressive joint disorder prevalent in elderly people, can cause chronic pain, joint stiffness, and even disability [30]. However, existing therapies are not effective enough to prevent the pathological progress or repair the cartilage degradation of OA [31]. Therefore, it is still imperative to deepen the understanding about the molecular mechanism of OA to identify new therapies. Previous studies have reported that IL-1β promotes the secretion of pro-inflammatory mediators and accelerates OA progression [32]. Therefore, many studies use IL-1β as the major stimulator for OA [33–35]. Herein, IL-1β was also employed to establish in vitro OA model.

USP25 has been reported to mediate inflammatory responses in human diseases. For example, USP25 deficiency attenuates microglia-mediated proinflammatory cytokine overproduction and synapse elimination in Alzheimer’s disease [36]. USP25 upregulation caused by LPS deubiquitinated HBO1 in THP-1 monocytes and human primary macrophages, and therefore mediated inflammatory gene transcription [10]. USP25 facilitated pro-inflammatory factor production and tight junction impairment via STAT3 pathway, causing exacerbated acute pancreatitis (AP) and AP-related organ injury [37]. In this study, we discovered that IL-1β stimulation increased the expression of USP25 in rat chondrocytes. More importantly, IL-1β treatment increased intracellular ROS level while impaired cell proliferation, suggesting that USP25 may contribute to OA progression.

Reactive oxygen species (ROS) could drive inflammatory responses and is a potent risk factor in OA progression [38]. Overproduction of ROS has been considered a possible therapeutic target in IL-1β-induced OA models [4, 39, 40]. Besides, pyroptosis, a pro-inflammatory programmed cell death mediated by NLRP3 inflammasome and caspase-1 activation [41, 42], participates in OA pathogenesis and pathological changes [43]. Moreover, it has been proved that matrix metalloproteases (MMPs) facilitate the degradation of extracellular matrix (ECM) and therefore mediate OA progression [44]. In this research, it was found that USP25 deficiency effectively attenuated the pyroptosis, ROS overproduction, NLRP3 inflammasome activation, and ECM degradation caused by IL-1β treatment. Furthermore, the promotive effect of USP25 overexpression on OA progression could be overturned by inhibiting ROS production, indicating that USP25 affects the development of OA in a ROS-dependent manner.

TXNIP, a key regulator in the redox system, inhibits thioredoxin (TXN) to produce cellular oxidative stress and disturb the activities of TXN-associated proteins [45]. The interaction between TXNIP and NLRP3 inflammasome has been implicated in various human diseases. For instance, Metformin inhibited TXNIP/NLRP3 interaction and ameliorated intestinal ischemia–reperfusion injury [46]. S-adenosylhomocysteine inhibition enhanced TXNIP-mediated oxidative stress and NLRP3 inflammasome activation, which aggravated podocyte injury and diabetic nephropathy [47]. TXNIP-mediated activation of NLRP3 inflammasome contributed to corticosterone-induced neuroinflammation [48]. Herein, it was identified that USP25 deubiquitinated TXNIP in IL-1β-induced chondrocytes, causing the upregulation of TXNIP protein expression. In addition, TXNIP depletion reversed the promotive effect of USP25 abundance on the pyroptosis, ROS production, NLRP3 inflammasome activation, and ECM degradation of IL-1β-induced chondrocytes. Consistently, inhibition of USP25 by AZ1 ameliorated articular damages and suppressed the pyroptosis, ROS level, and activation of NLRP3 inflammasome in OA rat model.

## Conclusion

This study illustrated that USP25 exacerbated OA in vitro and in vivo via the mediation of ROS production, pyroptosis, and ECM degradation through TXNIP/NLRP3 axis. This finding suggested that USP25 is a potent therapeutic target for OA treatment.

## Data Availability

The datasets used and/or analyzed during the current study are available from the corresponding author on reasonable request.
